# Medicines availability in Ecuador's public system (2015–2024): a policy design analysis of financing, public procurement, and governance

**DOI:** 10.1016/j.lana.2026.101399

**Published:** 2026-02-12

**Authors:** Alejandro Hernández-Luis, Guillaume Fontaine, José Luis Morales

**Affiliations:** The Comparative Policy Lab, FLACSO-Ecuador, Quito, Ecuador

**Keywords:** Health governance, Medicine shortage, Pharmaceutical supply, Public procurement, Policy design

## Abstract

Medicines shortages are a major health policy challenge in Ecuador and reflect inconsistencies in the architecture of public-sector medicines access policy. This Health Policy paper, grounded in the policy design framework, synthesises administrative records and policy documents (2015–2024) to show how budget contraction, volatile execution, and weak *ex ante* needs assessment hinder the translation of allocations into sustained availability. Procurement has shifted from a centralised electronic catalogue towards more fragmented purchasing modalities, while limited transparency in inventory management sustains uncertainty about needs and stock. Governance constraints, including institutional weakness, gaps in timely information, and extensive Constitutional Court oversight, further strain decision-making. Strengthening coherence across financing, procurement, information systems, and governance, alongside more stable leadership, is essential to translate formal commitments into reliable access to essential medicines.

## Introduction: medicine shortage as a policy problem

Inadequate access to essential medicines is widely characterised as a wicked problem for health systems.^1^ This gap contributes to millions of preventable deaths and exacerbates health inequalities. Explanations in the policy literature link the problem to contingent factors,^2^ including sectoral fiscal sustainability,^3^ deficits in transparency and corruption in public administration,^4^ limited capacities of regulatory and oversight bodies,^5^ the concentration of innovation and production in a small number of countries,^6^ limited experience with health technology assessment,^7^ and gaps in professional training that affect prescribing and treatment practices.^8^ In South America, these obstacles extend even to well-established medicines,^9^ generating episodes of shortage that erode public trust in health systems. This situation is particularly evident in Ecuador, where persistent weaknesses undermine sustained medicines availability.

Ecuador's near-total reliance on public health services, together with the rapid expansion of the private pharmaceutical market, underscores the urgency of assessing its medicines access policy framework. We engage with evidence on socioeconomic gradients in unmet need,^10^ the effects of market deregulation,^11^ the fragility of supply chains and import dependence,^12^ and regional purchasing and governance arrangements.^13^

Debates on medicine shortages take place in a highly politicised environment and often rely on fragmentary evidence. This paper grounds the discussion in a systematic empirical basis. We analyze the design of Ecuador's medicines access policy framework, with a focus on the conditions driving shortages, and propose feasible adjustments. Our approach draws on the policy design framework,^14^^,^^15^ and on the institutional analysis of policy instruments understood as state resources.^16^

## The policy problem of medicine shortages in Ecuador

Determining the magnitude of the problem of access to medicines in Ecuador is not straightforward due to weaknesses in the data sources. The national indicator of out-of-pocket health expenditure, for example, does not disaggregate the pharmaceutical component and therefore cannot measure households’ direct spending on medicines with precision. Moreover, it relies on the 2012 Household Income and Expenditure Survey and the 2014 Living Conditions Survey, which have not yet been updated; consequently, the data reported for 2016–2023 (30.6–33%) derive from pre-COVID-19 estimates^17^ rather than from direct measurements that would allow the effects of public interventions to be assessed. Nevertheless, the available sectoral information allows for characterising the context in which shortages occur.

The Ecuadorian pharmaceutical market is characterised by high external dependence, rapid retail expansion following deregulation, and rising prices. Imports between 2015 and 2022 ranged from USD 1.07 to 1.25 billion, peaking at USD 1.43 billion in 2021, while exports declined from USD 65 million to USD 35 million.^18^, ^19^, ^20^, ^21^ Over the same period, deregulation coincided with a marked expansion of the retail market, with the number of pharmacies increasing from 6000 to 12,964, equivalent to one pharmacy per 1307 inhabitants, roughly twice the OECD average.^22^, ^23^, ^24^ Price pressures also intensified, with the pharmaceutical products index rising from 99.9 to 121.8 (+21.9%).^25^ In this context of a saturated market and rising prices, dependence on the public sector heightens the risk of unmet need: in 2023, only 23.3% of the population had social insurance and 10% had private insurance, meaning that roughly two thirds (around 11.3 million people) relied on the Ministry of Public Health (MPH) as their principal provider.^26^

In this context, access to medicines enters the public agenda chiefly through social mobilisation. Since 2018, patients and health professionals have repeatedly protested against medicines shortages in public facilities.^27^, ^28^, ^29^, ^30^ These actions intensified during the pandemic and have remained prominent in media coverage, creating a highly conflictual environment and polarising the debate. Demands have focused, inter alia, on the lack of essential medicines and treatments for chronic, catastrophic and rare diseases, as well as interruptions to outsourced critical services (e.g., oncology and dialysis).^31^^,^^32^

In parallel, corruption acted as an aggravating factor. During 2020–2021—the so-called “other pandemic”^33^—more than 30 investigations were opened into irregularities and overpricing in the procurement of supplies and medicines, implicating senior officials of the public health system, legislators and even a former president.^34^^,^^35^ Although few cases culminated in convictions, extensive media coverage consolidated perceptions of opportunism in public purchasing and deepened public distrust.

Faced with social pressure and accumulating distrust, the MPH was compelled to respond. In the absence of unit-level public data on availability, it acknowledged that “shortage is not a perception but a fact”^36^ and declared two health emergencies. The 2022 decree recognised shortages since 2020 and an availability level of 69% against a target of >90%. The September 2025 decree reported continued difficulties since 2023 and a rolled-over budget with “non-executable sources, preventing its effective use”.^37^ Although the resolution did not specify a national availability percentage, the MPH reported hospital levels of 18%–77%.^38^

Occasionally, these allegations have triggered horizontal accountability mechanisms. In 2019 and 2023, the National Assembly censured the Ministers of Health for improper involvement in revisions of the National Basic Medicines Formulary (NBMF), the purchase of expired medicines, and failure to prevent shortages.^39^^,^^40^ The Office of the Comptroller General documented recurrent irregularities, including deficient inventory management, the absence of market studies, and inadequate procurement planning.^41^ In addition, more than 100 rulings by the Constitutional Court have been issued, some of which have influenced the design of access-to-medicines policies.^42^

While other challenges persist, such as the circulation of falsified or substandard medicines^43^^,^^44^ and territorial inequalities in access,^45^ the immediate priority is to restore the availability of medicines in the public system. This priority directly affects treatment continuity and the system's legitimacy.

## Methods

This Health Policy paper draws on an in-depth, single-country case study of Ecuador, combining a narrative review of policy and regulatory documents with a descriptive use of existing administrative records. We brought together (i) an exhaustive review of policy documents, health regulations and legal frameworks on access to medicines and (ii) administrative data from the Ministry of Economy and Finance, the National Public Procurement Service (SERCOP), the NBMF, and current norms governing access to medicines and public purchasing. We selected the period 2015–2024 because, since 01 January 2015, public procurement data have been available in Open Contracting Data Standard open format, enabling systematic analysis.^46^ The analysis is guided by the “instrumentation” function of the policy design framework, which interprets the resources the state allocates to addressing a public problem as expected empirical manifestations of the policy process (Panel 1). Quantitative information is used descriptively to contextualise the policy discussion and does not involve formal hypothesis-testing or impact evaluation.

Panel 1 **Policy instruments as empirical observations of the policy process**

The policy-design framework conceives design as the attempt to articulate a coherent set of instruments that links problem definition to public intervention.^47^ To classify these instruments, we draw on Christopher Hood’s typology of governmental resources—nodality, authority, treasure, organisation (NATO)^48^—and adapt it functionally to four categories used in this study: finance, information, regulation, and administrative organisation. The correspondence with the original scheme is preserved.

• Finance (treasure): resources through which the state allocates or transfers money, or other fungible goods, to public or private actors to enable action.
• Information (nodality): resources that allow the state to generate, concentrate or disseminate information required to steer behaviour or coordinate actors.
• Regulation (authority): resources expressing a legal or official power to require, prohibit, enable or guarantee specified behaviours.
• Administrative organisation (organisation): resources that mobilise structures, entities and government personnel to execute decisions directly.

Understood in this way, instruments are the basic mechanisms by which the public sector seeks to influence society and the market. In this study, we use this classification to identify, across legal and policy documents, the budget, and procurement records, the concrete traces of the policy process. A forward mapping of the instrument mix shows whether the state responded to the problem with a coherent set of measures or, instead, with overlapping and fragmented interventions.

To describe public expenditure on medicines, we used monthly data from the “Budgetary Classification of Revenues and Expenditures of the Public Sector” of Ecuador's General State Budget. Specifically, we examined the current expenditure (530,809) and investment expenditure (730,809) lines for medicines and considered all four budget modalities of the state. All amounts are expressed in United States dollars (USD).

For procurement processes, we used SERCOP open-data records to identify procedures that included the term “medicines” in their description. For low-value procedures not available on the open-data portal, we obtained an anonymised database directly from SERCOP (incident No. 2025019780; response dated 17 March 2025). The combined dataset covers January 2015 to December 2024 and captures procurement procedures related to medicines recorded in the national system. Because some records aggregate purchases under the general heading “medicines” without item-level detail, descriptive estimates based on these data should be interpreted as conservative.

Using R, we extracted the process title, award amount, award date and procurement modality and produced simple descriptive summaries of total medicines expenditure by modality to support the narrative analysis. No new data were generated and no individual-level information was accessed; all empirical references rely on published or publicly available administrative sources.

## Inefficient public expenditure and diversification of procurement modalities (finance)

**Fig. 1:**
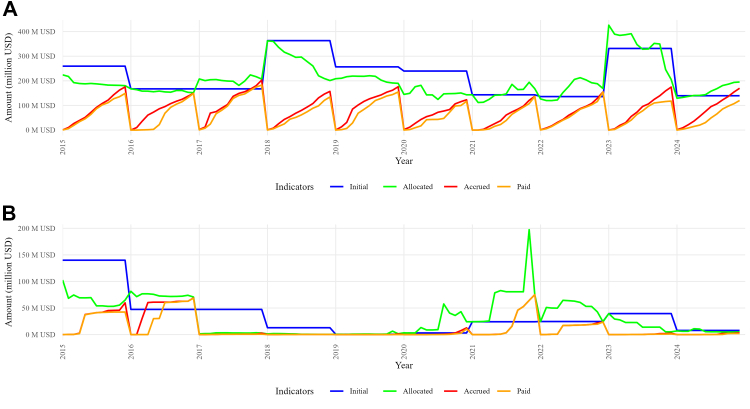
**Evolution of current and investment public spending in medicines in Ecuador, 2015–2024.** Panel A) current spending; Panel B) investment spending. *Source*: Authors' elaboration based on items 530,809 (Current Goods and Services—Medicines) and 730,809 (Capital Goods and Services—Medicines) from the Budgetary Classification of Public Sector Income and Expenditure. Available from: https://www.finanzas.gob.ec/ejecucion-presupuestaria/.

In low- and middle-income countries, the availability of medicines depends on the execution of public spending on health.^49^^,^^50^ This underscores the importance of budget planning and execution as core financing instruments within the medicines access policy framework. In Ecuador, current public spending on medicines consists primarily of direct public procurement, whereas investment public spending is directed towards acquiring physical assets and developing infrastructure, as well as production, storage, and distribution logistics capacities. Fig. 1 summarizes the evolution of this expenditure over the period 2015–2024.^51^

Our examination of available budget and expenditure figures indicates a threefold pattern. First, there is a systematic discrepancy between the initial budget and the expenditure recorded each year. This budgetary gap was most pronounced in 2015, 2018–2020, and 2023 for current expenditure, and in 2015, 2017, and 2023 for investment expenditure. This pattern is consistent with the absence of robust *ex ante* needs-assessment methods and an optimistic bias in resource allocation, partly shaped by political incentives.

Second, budget execution is highly erratic. The ratio of executed to codified current expenditure ranges from 78.1% in 2018, to 85% in 2023–2024 and to 98% in 2015–2017. Likewise, the execution of investment expenditure ranges from 48% in 2023, to 55.5% in 2018, to 90% in 2015–2017 and to 100% in 2021–2022. Such volatility undermines the government's capability to translate state resources into a sustainable supply of medicines.

Third, the progressive contraction of public spending in medicines has led to a continuous decline in final execution (cumulative current and investment expenditure), falling from USD 235 million to USD 135.7 million between 2015 and 2020, followed by a temporary recovery to USD 212.3 million in the second year of the COVID-19 pandemic, and stabilising afterwards at USD 173 to 178 million. In other words, the exceptional mobilisation of state resources during the pandemic did not reverse the trend of suboptimal budget execution, despite the increased long-term demand for medicines, which reveals recurring policy implementation gaps.

**Fig. 2:**
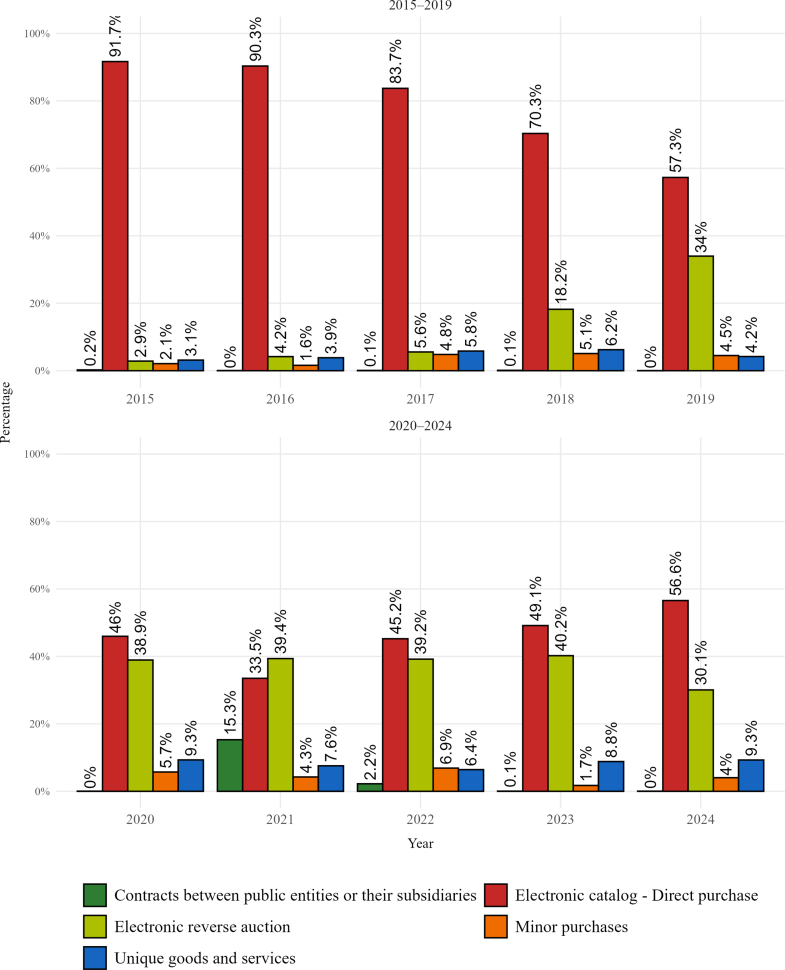
**Annual share of public procurement expenditure for medicines by modality in Ecuador, 2015–2024.***Source*: Authors' elaboration based on SERCOP (Servicio Nacional de Contratación Pública) open data (Available from: https://datosabiertos.compraspublicas.gob.ec/PLATAFORMA/datos-abiertos); including minor purchases.

Disaggregated data show that budget execution across contractual modalities changed markedly over the reference period (Fig. 2).^52^ Because medicines procurement is recorded under current expenditure, this spending represents the largest component of the state budget for the acquisition of medicines through public procurement, which is implemented through five modalities described below.

Direct purchasing through the national electronic catalogue is the primary modality; it accounted for 91.7% of total procurement in 2015 but declined to 33.5% by 2021 (Fig. 2). Meanwhile, this regime was increasingly replaced by reverse auctions conducted outside the electronic catalogue (Fig. 2).

The remaining three regimes comprise minor purchases, unique goods and services, and contracts between public entities or their subsidiaries (institutional purchases). Minor purchases remained marginal, accounting for between 2.1 and 4% of the total, except in 2022, when it reached 6.9% (Fig. 2). Institutional purchases were similarly residual (less than 0.2% in 2015) and declined thereafter, except during the pandemic, when they rose to 15% (Fig. 2). Notably, the unique goods and services regime increased from 3.1% (2015) to 9.2% (2024) (Fig. 2). This increase indicates greater reliance on sole providers in the local market, which raises concerns from a corruption-control perspective.

In summary, these trends confirm a shift from a model centred on the electronic catalogue to a more diversified procurement arrangement, marking a substantial institutional change. To interpret the observed variations and tensions in budget execution, however, it is necessary to consider how financing interacts with the other policy instruments. Rather than an isolated development, this diversification, together with persistent weaknesses in budget execution, points to broader inconsistencies in medicines access policy design. Analysing how these instruments operate in combination is therefore essential to explaining why resources and formal commitments have not translated into sustained medicines availability in Ecuador's public system.

## Shortcomings in Ecuador's medicines access policy design: planning without operationalisation for essential medicines (information)

Two informational instruments are particularly relevant to Ecuador's policy framework for access to medicines: the National Medicines Strategy (NMS) and the NBMF. The former is the official document that presents the government's vision and sets out the basic guidelines to ensure access to medicines. The latter comprises a list of essential medicines, aligned with epidemiological, cost-effectiveness, and supply sustainability criteria. This section examines the genesis and scope of this Strategy, as the main planning instrument, before assessing the operational role of the Formulary, as a complementary instrument of information.

The first NMS was adopted in 2007 and remained the principal formal planning instrument until 2017. This document included a concise description of the policy problem, focusing on the high health costs borne by the poorest populations. It also highlighted the low share of generic medicines, market concentration among a handful of stakeholders, the urban–rural gap in access to pharmacies, and the discrepancy between consumer practices and social needs. Based on this diagnosis, the government's strategy aimed to achieve universal access, improved quality, rational use, increased national production, and greater transparency in public procurement. Yet these declarations amounted to little more than wishful thinking, as they failed to specify the operational mechanisms, instruments, indicators, and goals necessary for assessing and evaluating the strategy.

A new Strategy was adopted in 2017,^53^ and it remains the sole orientation document for the government's medicines access policy. It provides a bureaucratic and legal justification that, while acknowledging Ecuador's international obligations, insists on aligning the medicines access policy framework with constitutional principles, the organic laws on health and the national health system, the legislation on generic products for human use, and the five-year National Development Plan (2013–2017).

The diagnosis provided in the 2017 Strategy is more detailed and precise than in the 2007 Strategy. It begins by acknowledging the unavailability of certain essential medicines and compounded preparations listed in the NBMF. It also describes the mandatory marketing authorisation and regulatory control procedures, but does not address substandard and falsified medical products or weaknesses in quality-assurance systems across manufacturers. Further, it underscores the need to assess the therapeutic value of medicines, to improve the selection process for the Formulary, and to evaluate health technologies. However, this section lacks the disaggregated data necessary to enable a precise description and a clearer understanding of the policy pathways prioritised by the government.

The 2017 Strategy also offers strategic guidance that includes goals and indicators, giving the policy design the veneer of evidence-based decision-making. Yet from the outset, its practical effectiveness is undermined by the absence of a baseline and any benchmark for monitoring progress; consequently, the annual objectives prove unattainable.

Hence, in the absence of formal protocols in the evaluation methodology, the National Institute of Statistics did not incorporate the indicators of the NMS into its accounts or any other official records. In fact, the 2017 Strategy has never been evaluated owing to the lack of basic information with which to compare its outcomes. In other words, this was a fundamental flaw in strategic planning from the very beginning. This planning model, which lacks operationalisation, ultimately generated inconsistencies in the policy design by preventing any meaningful evaluation or adjustment.

The other key informational instrument—the NBMF—stems from the WHO Essential Medicines List (EML). It has been updated 11 times since its original publication in 1986, with notable revisions in 2014, 2019 and 2022.^54^, ^55^, ^56^ The formulary is based on the national epidemiological profile and aims to address basic health needs. It seeks to optimise the purchase and distribution of high-quality medicines, while promoting their rational use and reducing costs for both the health system and the population.^57^ Yet, the WHO reports that, in low- and middle-income countries, the availability of these medicines in pharmacies ranges from 8% to 41%,^58^ which raises questions about the effectiveness of this instrument and reinforces the need for further evaluation.

Over time, the NBMF has followed a similar trajectory to the EML and currently contains roughly twice as many line items as its first edition. As of 2022, it listed 484 active ingredients and 671 pharmaceutical products. However, there are still no direct indicators routinely used to assess its effectiveness or to determine whether this sustained increase in scope actually meets the health needs of the Ecuadorian population. Existing data systems do not systematically link Formulary updates to procurement decisions, stock levels, prescribing patterns or household expenditure on medicines. As a result, it is not possible to establish whether changes in the Formulary have translated into improved access in practice, and the instrument remains largely unevaluable.

Taken together, the NMS and the NBMF illustrate a planning approach without operationalisation within Ecuador's policy framework for access to medicines in the public system. Both instruments are formally aligned with constitutional principles, sectoral legislation and international guidance, but they lack the operational mechanisms, indicators and information systems required for their implementation, monitoring and evaluation. In the absence of these basic evaluative tools, the government's access-to-medicines policy framework cannot be meaningfully adjusted in light of its results.

## Judicial and regulatory shortcomings (regulation)

Regulation instruments of Ecuador's medicines policy consists of a complex framework that comprises the Organic Law of Public Health alongside a body of technical and procedural norms. The two most significant instruments are (i) the 2020 Constitutional Court ruling (case 679-18-JP/20), which establishes binding criteria on the availability and accessibility of medicines, and (ii) the Regulation on Medicines Supply, Medical Devices, and Administrative and Financial Control Management, which defines operational processes for procurement, storage, and inventory oversight within the public health system.

Case 679-18-JP/20 arose from social claims based on several consolidated constitutional rights actions concerning the denial or unavailability of medicines for catastrophic or highly complex diseases. The Constitutional Court selected the case for review of constitutional guarantees to harmonise legal standards, address administrative inaction, and set binding benchmarks for availability and accessibility. From an institutional perspective, the Court is the supreme oversight agency, responsible for reviewing the compatibility of public policies with the Constitution. However, for the sake of the due process, judges are not supposed to design or constrain the government in designing public policies. In that instance, the Court instructed the MPH to design a special policy for catastrophic and highly complex diseases,^59^ a step that may be perceived as encroaching on the policy-making prerogatives of the executive.

More importantly, the Court ordered the government to adapt the NMS and to incorporate a set of indicators it had devised. These drew mainly on the 1988 Protocol of San Salvador,^60^ an Organization of American States instrument that addresses a broad spectrum of economic, social and cultural rights. While pertinent to a rights-based approach, the Protocol offers high-level guidance and does not operationalise access to essential medicines. For monitoring and evaluation, internationally standardised metrics provide greater specificity and comparability—e.g., SDG indicator 3.b.3,^61^ established procurement and supply-chain indicators,^62^ accountability/oversight metrics,^63^ and the WHO measures related to essential medicines for universal health coverage.^64^ Cross-referencing these tools would enhance alignment with global practice and facilitate benchmarking; the Court judgment does not explicitly cite them.

The scope of these indicators extends beyond medicines availability and accessibility, encompassing specific metrics on palliative care and broader measures such as the implementation of a unified electronic health record across the public health system. For instance, the Court adopts a broad reading of the constitutional principle of progressive public spending on health (up to 4% of the Gross Domestic Product), which may be understood as implying a non-regressive constraint on medicines spending.^65^ In several cases, the indicators do not clearly specify data sources or articulate how they would contribute to monitoring and progress.

So broad a perspective downplays the central issue of medicines access, and constrains the government's role in policy design. It is also odd that the constitutional ruling orders the design of a policy for catastrophic and highly complex diseases within the framework of the NMS. The Strategy could instead guide the creation of disaggregated budget allocations for medicines both within and outside the NBMF, or establish a monitoring system for medicines dispensed beyond the Formulary.

Lastly, the 2020 ruling establishes no timeline nor specific deadlines for implementation, which goes against the basic principles of policy design. This omission further undermines public accountability and helps to explain why, by late 2025, the aspects of the ruling examined here had not been implemented.

The Regulation on Medicines Supply, Medical Devices, and Administrative and Financial Control Management is the operational instrument of regulation for medicines selection and public procurement. However, it suffers from serious flaws, such as the lack of oversight mechanisms to ensure a coherent policy implementation. Most of the responsibility for medicines sletelection is assigned to therapeutic committees and pharmacists. The document lets them elaborate lists based on the NBMF, without any validation or review process by the national health agency.

This discretionary capacity raises questions about how the government makes sure that the aggregation of these lists accurately respects the guidelines of that Formulary. Not only does the demand planning rely on estimates based on historical consumption rather than a robust forecasting system, but the selection is not even overseen by the national agency in charge of evaluating and standardising these projections.

Regarding public procurement, each establishment sets its own minimum stock levels and reorder points to trigger contracting procedures, which can lead to significant variation in both lead times and procurement volumes across facilities. With respect to inventory oversight, although the Regulation requires each warehouse to use a certified information technology system, it does not mandate a unified management platform. This hinders the consolidation of regional or national monitoring and prevents the early detection of shortages.

The fact that the Regulation assigns so much responsibility to therapeutic committees and pharmacists is inconsistent with the diagnosis provided by the 2017 NMS. This document had identified their institutional weaknesses, and noted that delegation to the local level created more confusion than solutions, owing to high turnover among the committees and their stakeholders' limited training and technical capacity to appraise scientific evidence.^53^ Actually, one of the Strategy's priorities was to restructure and strengthen these agencies. Yet, in the absence of evidence of such improvements, the Regulation nevertheless places most oversight in their hands. Once again, the regulatory instruments expose the inconsistency of the policy design.

## Lack of coordination in institutional arrangements (administrative organisation)

The institutional design of the NMS relies on the National Health System, which includes two principal coordinating bodies—the National Health Council (CONASA) and the Integrated Public Health Network (RPIS)—and engages with all stakeholders in the health sector. Although it is intended to operate under the stewardship of the MPH, coordination challenges and inter-agency competition persist.

The CONASA enjoys administrative and financial autonomy. It was created in 1980 and is enshrined in the 2002 Organic Law of the National Health System.^66^ It was conceived as a consultative body for all actors within the System and was tasked with supporting the Ministry in policy design. With respect to medicines, it works with the National Commission on Medicines and Supply to update the NBMF.

A central source of the coordination problems lies in normative and hierarchical tensions between the CONASA and the MPH. The 2008 Constitution and the 2006 Organic Health Law assign stewardship of public health to the MPH, yet CONASA claims the status of supreme body of the National Health System, drawing on the 1998 Constitution and the 2002 Organic Law of the National Health System.^67^

These normative tensions are further exacerbated by a fragile coordination arrangement between the MPH and CONASA. Over the ten-year period analysed, 12 different ministers held office,^68^ implying an average tenure of less than one year; this turnover accelerated during the COVID-19 pandemic, when four ministers were appointed in 2021 alone, in stark contrast with the stability of CONASA's Executive Directorate. Such instability prevents the MPH from completing basic budgetary and planning cycles and leads to constantly shifting agendas and political teams. In this context, it is to be expected that a more stable technocratic apparatus such as CONASA's will gain relative weight and progressively absorb key functions in agenda-setting and technical approval, placing itself in a privileged position to block, dilute, or redirect ministerial initiatives.

This imbalance is reinforced by an accountability gap that further weakens inter-institutional coordination. While the MPH has been subject to transparency and access-to-information requirements since the 2004 Transparency Law, CONASA only became formally required to comply with transparency provisions in September 2024.^69^ As a result, even basic information—such as the historical institutional organigram or the composition and rules of procedure of the expert bodies responsible for the NBMF—has been difficult to trace. This opacity is particularly problematic given that the Formulary is a highly sensitive policy instrument in which potential conflicts of interest among industry, prescribers, and regulators demand rigorous public oversight. In practice, the combination of weak coordination, asymmetric transparency, and limited scrutiny of conflicts of interest creates parallel decision-making circuits between the MPH and CONASA and turns the latter into a de facto veto player vis-à-vis the ministry, making it difficult to sustain a coherent and accountable long-term medicines policy.

The RPIS, by contrast, was referenced in the 2008 Constitution but formally established in 2012 to coordinate public healthcare providers with the social security institutions.^70^ This entails formally aligning the agendas of the ministries of Health, Interior and Defence, on the one hand, and the three social security schemes—general, police and armed forces—on the other. However, coordination is constrained by the financial and managerial autonomy of the social security subsystems and by marked asymmetries in their purchasing power. Operated through inter-institutional agreements and specialised units within each entity,^70^, ^71^, ^72^ the effectiveness of coordination is therefore largely contingent on the willingness of the actors involved.

Operationally, the RPIS has introduced mechanisms for joint procurement and for the development and interoperability of information systems, though these remain limited. The most prominent arrangement was the corporate reverse auction through the electronic catalogue; however, as noted above, this has gradually been supplanted by ad hoc purchasing.

Moreover, the model that combined joint procurement with a unified platform for the national medicines inventory was never implemented and was suspended in 2022. As a result, purchasing processes remain fragmented and no IT system provides precise data on what the Network has in stock. This affects government capacity to anticipate shortages and adopt timely responses to meet public-system needs.

## Conclusions and recommendations

This Health Policy paper shows that recurrent shortages in Ecuador's public system are closely linked to fragmented medicines access policy design. In particular, inconsistencies across the policy instrument mix—financing, information, regulation, and administrative organisation—undermine the system's capacity to translate resources and formal commitments into sustained medicines availability.

In practice, this fragmentation is reflected in sharp budget contraction, erratic financing along the medicines supply chain, and the absence of robust *ex ante* needs assessment methods. It has also coincided with a shift from a centralised e-catalogue to fragmented purchasing that disperses public demand, and whit the absence of joint procurement and interoperable information systems.

Institutional arrangements further weaken policy coherence. The MPH has limited capacity to implement the Strategy amid encroachment by the Constitutional Court and CONASA. The RPIS has not fulfilled its coordinating role. Hence, the system lacks a coherent procurement system, an integrated logistics chain, and compatible inventory registers.

The lack of reliable baseline data limits evaluation and course correction, while opacity of the information systems prevails. At the same time, weak coordination, institutional layering, and veto players delay government decision-making. The resulting pattern of erratic public management undermines responsiveness to social needs and fuels public frustration. Ultimately, inconsistencies in policy design are not merely technical; they also carry political consequences by eroding trust and stability.

Our descriptive use of budget execution and procurement records, together with the institutional analysis of policy instruments, should be interpreted with three caveats. First, procurement databases do not capture all purchases, so the figures reported are conservative. Second, our assessment relies on administrative data and lacks clinical or financial outcome indicators, which prevents us from estimating effects on health outcomes or costs. Third, out-of-pocket expenditure associated with shortages is likely underestimated. Even so, the evidence indicates that fragmented policy design is a structural determinant of access to medicines. On this basis, we propose a focused set of priorities to address failures in financing, operational coordination, and governance.

We propose three strategic priorities to address these failures. First, to reinforce financial planning, the government should develop a robust *ex ante* methodology to estimate medicines procurement needs and secure stable budget allocations aligned with the epidemiological profile and consumption patterns. Second, to consolidate purchasing and information systems, it should re-establish a centralised joint-procurement mechanism to reduce reliance on ad hoc practices and implement a unified national information technology platform for the medicines inventory to enable real-time monitoring and early shortage detection. Third, to clarify governance and accountability, it should ensure stability in the leadership of the MPH by reducing ministerial turnover to support consistent implementation, medium-term planning, and continuity of strategic reforms; resolve the mandate ambiguity between the MPH and CONASA to ensure clear government leadership; limit judicial overreach by embedding technical, evidence-based indicators in policy design; and strengthen therapeutic committees through training and professional stability while ensuring national oversight of their decisions.

Ecuador's difficulties in sustaining access to essential medicines do not stem from a lack of strategies or legal frameworks. Rather, poorly aligned instruments, limited operational capacity, and inconsistent oversight constrain sustained availability. Strengthening state capacity is essential to implement decisions, coordinate institutions, and generate timely information across the supply chain. The priority is to rebuild coherence, restore leadership, and ground policy in transparent, evidence-based instruments so commitments translate into guarantees in practice.

## Contributors

Alejandro Hernández-Luis led the conceptualisation of the study, developed the methodology, and managed the project. Alejandro Hernández-Luis drafted the original manuscript and contributed to subsequent revisions. Guillaume Fontaine contributed to the methodological development, provided supervision throughout the project, and co-drafted the original manuscript; Guillaume Fontaine also contributed to manuscript revisions. José Luis Morales co-drafted the original manuscript, led the visualisation of figures and tables, and contributed to manuscript revisions. All authors reviewed and approved the submitted version of the manuscript and agree to be accountable for all aspects of the work. Alejandro Hernández-Luis made the decision to submit the manuscript.

## Use of artificial intelligence

The authors used generative artificial intelligence (ChatGPT, OpenAI) to support English-language editing and improve clarity in selected passages of the manuscript. The authors reviewed and edited all AI-assisted text and take full responsibility for the content.

## Declaration of interests

Alejandro Hernández-Luis reports paid consultancy work for the Pan American Health Organization (PAHO/WHO), supporting Ecuador's Ministry of Public Health, within the past 36 months, outside the submitted work. This manuscript is based exclusively on publicly available sources, all of which are cited; it does not use any non-public data, consultancy deliverables, or materials produced under that contract. Guillaume Fontaine and José Luis Morales declare no competing interests.

## Appendix A Supplementary data

Translated summary
