# Supplementary material for: Medicines availability in Ecuador's public system (2015–2024): a policy design analysis of financing, public procurement, and governance
Source: Lancet Reg Health Am. 2026 Feb 12;56:101399. doi: 10.1016/j.lana.2026.101399 (PMC12925043; doi:10.1016/j.lana.2026.101399)
Supplement: Translated summary [file mmc1.docx]

**Editorial disclaimer**

The translation of the Summary was submitted by the authors, and we reproduce it as supplied. It has not been peer reviewed. Our editorial processes have only been applied to the original version in English, which should serve as a reference for this manuscript.

**Resumen**

El desabastecimiento de medicamentos constituye un desafío importante de política de salud en Ecuador y refleja inconsistencias en la arquitectura de las políticas de acceso a medicamentos del sector público. Este artículo de políticas públicas, basado en el enfoque de diseño de políticas, sintetiza registros administrativos y documentos de política (2015–2024) para mostrar cómo la contracción presupuestaria, la ejecución volátil y una débil evaluación ex ante de necesidades dificultan la traducción de las asignaciones en disponibilidad sostenida. Las compras públicas han transitado desde un catálogo electrónico centralizado hacia modalidades de adquisición más fragmentadas, mientras que la limitada información en la gestión de inventarios mantiene la incertidumbre sobre las necesidades y los niveles de stock. Las restricciones de gobernanza, incluida la debilidad institucional, las brechas en la disponibilidad oportuna de información y la supervisión de la Corte Constitucional, tensionan aún más la toma de decisiones. Fortalecer la coherencia entre financiamiento, compras públicas, sistemas de información y gobernanza, junto con un liderazgo más estable, es esencial para traducir los compromisos formales en un acceso confiable a medicamentos esenciales.
